# THE USE OF INVOLUNTARY TREATMENT AMONG NURSING HOME RESIDENTS IN THE NETHERLANDS: A CROSS-SECTIONAL STUDY

**DOI:** 10.1093/geroni/igae098.2803

**Published:** 2024-12-31

**Authors:** Guido Biesmans, Niels Hameleers, Jan Hamers, Michel Bleijlevens

**Affiliations:** Maastricht University, Maastricht, Limburg, Netherlands; Maastricht University, Maastricht, Limburg, Netherlands; Maastricht University, Maastricht, Limburg, Netherlands; Maastricht University, Maastricht, Limburg, Netherlands

## Abstract

Several studies indicate that care-dependent older adults with cognitive impairments, living in a nursing home, are at risk of care without their consent, referred to as ‘involuntary treatment’. This includes the use of physical restraints (e.g. belt restraint or bedrails), off-label use of psychotropic medication (e.g. anti-depressants, sedatives) and non-consensual care (e.g. forced hygiene, hiding medication). To explore the prevalence and associated factors of involuntary treatment in nursing home residents with cognitive or functional impairment we conducted a cross-sectional study in the southern part of the Netherlands using association analyses. Involuntary treatment was measured using the International Prevalence Measurement of Care Quality (LPZ). In addition, sociodemographic data were collected from residents, including gender, age, functional or cognitive impairment, diagnosis of dementia, confusion, aggression and dependency in activities of daily living. Involuntary treatment was used in 30% of the total sample. Among the residents who received at least one form of involuntary treatment, physical restraint was most commonly used (92%), followed by off-label use of psychotropic medications (20%) and non-consensual care (10%). Involuntary treatment was most common among residents with cognitive impairments (38%). Within the group of residents with functional impairment, the prevalence was 20%. Cognitive impairment and dependency in activities of daily living were strongly associated with involuntary treatment. Involuntary treatment is often used among nursing home residents with cognitive impairment. It also shows that residents with functional impairments are at risk. Future research should focus on understanding and preventing inappropriate involuntary treatment in nursing homes.

